# Incorporating demographic stochasticity into multi-strain epidemic models: application to influenza A

**DOI:** 10.1098/rsif.2008.0467

**Published:** 2009-01-21

**Authors:** Pavlo Minayev, Neil Ferguson

**Affiliations:** MRC Centre for Outbreak Analysis and Modelling, Department of Infectious Disease Epidemiology, Faculty of Medicine, Imperial College of Science, Technology & Medicine, Norfolk Place, London W2 1PG, UK

**Keywords:** multi-strain model, population dynamics, transient strain-transcending immunity, cross-immunity, strain diversity, influenza dynamics and evolution

## Abstract

We develop mathematical models of the transmission and evolution of multi-strain pathogens that incorporate strain extinction and the stochastic generation of new strains via mutation. The dynamics resulting from these models is then examined with the applied aim of understanding the mechanisms underpinning the evolution and dynamics of rapidly mutating pathogens, such as human influenza viruses. Our approach, while analytically relatively simple, gives results that are qualitatively similar to those obtained from much more complex individually based simulation models. We examine strain dynamics as a function of cross-immunity and key transmission parameters, and show that introducing strain extinction and modelling mutation as a stochastic process significantly changes the model dynamics, leading to lower strain diversity, reduced infection prevalence and shorter strain lifetimes. Finally, we incorporate transient strain-transcending immunity in the model and demonstrate that it reduces strain diversity further, giving patterns of sequential strain replacement similar to that seen in human influenza A viruses.

## 1. Introduction

Modelling of the transmission of multi-strain pathogens to date has either used compartmental deterministic frameworks (Andreasen *et al*. 1997; Gupta *et al*. 1998; Gog & Grenfell 2002) or large-scale individually based simulations (Ferguson *et al*. 2003; Koelle *et al*. 2006). While the former retain sufficient simplicity to allow (quasi-) analytical insight into system dynamics, they cannot satisfactorily capture the intrinsically stochastic nature of strain creation (via mutation or recombination) and extinction (Minayev & Ferguson 2008). Simulations can capture such processes more realistically, and past work has shown that stochasticity plays a key role in explaining the evolution of influenza A in humans (Ferguson *et al*. 2003; Koelle *et al*. 2006), but in this case structural and computational complexity means that insight into key drivers of the dynamics can be compromised. Here we try to bridge that gap, by introducing discrete strain extinction and generation (i.e. mutation) processes into previously developed deterministic model frameworks (Minayev & Ferguson 2008). Our aim is to find the minimally complex model capable of giving an at least qualitatively realistic description of the evolutionary dynamics of influenza, i.e. a model giving relatively low standing diversity of extant strains despite a very large potential diversity, together with frequent sequential strain replacement (Bush *et al*. 1999; Smith *et al*. 2004).

Deterministic epidemic models governed by a set of nonlinear differential equations have the well-recognized limitation that, as all the dynamical variables are real valued, a strain, once it becomes established, never goes completely extinct, and instead continues to infect a (perhaps vanishingly) small proportion of the population. As a result, very long-period epidemic cycles of individual strains can occur, despite trough prevalences of 10^−20^ or less. Equilibrium strain diversity can therefore be unrealistically high. In order to provide better quantitative estimates for disease persistence or elimination, more biologically realistic models need to take into account the probability of extinction of the disease as soon as the proportion of infectious falls to low levels. We therefore extend the fully deterministic compartmental model explored in Minayev & Ferguson (2008) by introducing an extinction threshold according to the following algorithm: if there is only one individual infected with a particular strain in the population and no transmission takes place during the mean infectious period, this strain is eliminated from the simulation (i.e. the proportion infectious with this strain is set to 0). This modification of course introduces the notion of population size into the model.

Another factor that is important to a realistic description of the process of emergence of new strains is the discrete and stochastic nature of mutation. Therefore, we model mutation stochastically, by picking the number of individuals who experience mutations during their infectious period from a Poisson distribution. We assume, as we did for the deterministic model, that mutation only causes a one allele change and that after mutation hosts become infectious with the mutated strain.

We first develop the model, before exploring how its dynamics differ from the deterministic model without extinction or stochastic mutation. We study model variants with and without transient strain-transcending immunity of the type first postulated in Gupta *et al*. (1998) to explore to what extent such immunity is necessary to reproduce influenza-like evolutionary dynamics.

## 2. Model definition

We consider pathogen strains to be sequences of *N*_L_ antigenic loci with each locus able to be occupied by one of *N*_A_ alternative alleles, giving a total of 
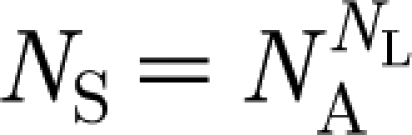
 different combinations. Infection, recovery from infection, birth and death are described deterministically, and are governed by the same equations as in our previous work (Minayev & Ferguson 2008). Namely, the dynamics of the population susceptible to strain *i* is described by2.1
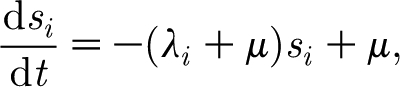

where *λ*_*i*_=*βy*_*i*_ is the force of infection of strain *i*; *μ* is the birth and death rate (births balance deaths); *β* is the transmission coefficient; and *y*_*i*_ is the proportion of the population infectious with strain *i*.

The dynamics of 
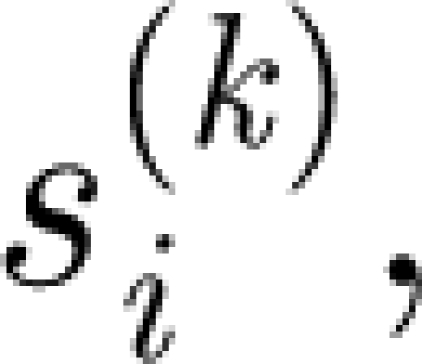
 the proportion of the population susceptible to any strain that shares alleles with with *i* but contains not more than *k* alleles different from *i*, is given by2.2
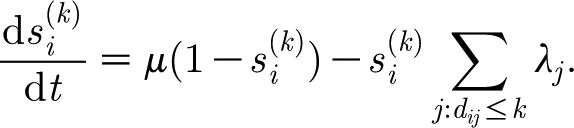



In the sum in (2.2), only those strains *j* whose genetic distance from strain *i* does not exceed *k* are considered. Index *k* ranges from 1 to (*N*_L_−1) and by definition we put 
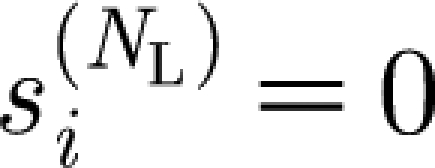
 and 
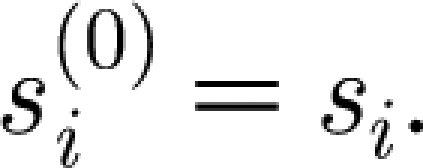
.

We assume, as we did in Minayev & Ferguson (2008), that, upon infection with strain *i*, a host gains a partial protection against any strain *j*. Cross-immunity acts to reduce the transmission success by a factor (1−*γ*(*d*_*ij*_)), where *γ*(*d*_*ij*_) is the cross-immunity function that we suppose linearly decreases with the genetic (Hamming) distance *d*_*ij*_ between strains *i* and *j*
2.3
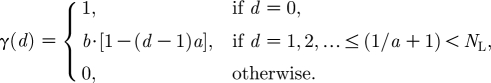

For a more detailed discussion of the deterministic equations (2.1) and (2.2) and the function (2.3) describing the cross-immune response, the interested reader can see our previous paper Minayev & Ferguson (2008).

The dynamics of the infectious compartments is governed by2.4
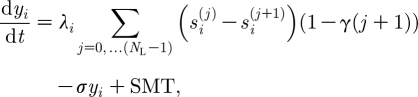

where *σ* is the recovery rate and SMT is a stochastic (Poisson) term representing the mutation process. Mutation is modelled at the level of individual hosts and we assume that, as a result of mutation, a host becomes infectious with a new strain. The rate of the corresponding Poisson process is 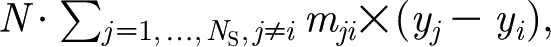 where *N* is the total population size and *m*_*ji*_ is the rate characterizing mutation *j*→*i* (we assume only one-point mutations: *m*_*ji*_=*m*_*ij*_=*m* if *d*_*ij*_=1, otherwise *m*_*ji*_=0).

The dimensionality of the model, 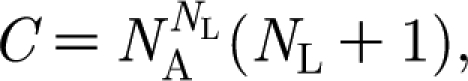 is the same as in the deterministic case we studied.

We can also add transient strain-transcending immunity to the model, as hypothesized in Ferguson *et al*. (2003) and described in Minayev & Ferguson (2008). We assume that a host becomes temporarily completely immune to all strains upon infection but then loses this immunity at a rate *γ*. Dynamics of the temporarily immune populations required to model this is described by the equations2.5
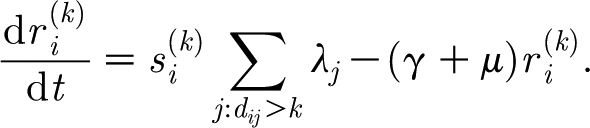

Equations (2.1) and (2.4) retain their form and (2.2) is modified to include the term describing population returning from the temporarily immune to the susceptible state2.6
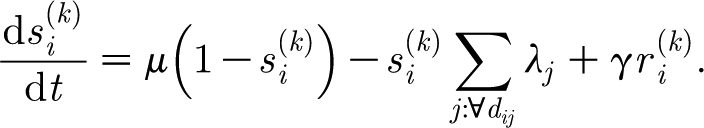

The dimensionality of this extended model, 
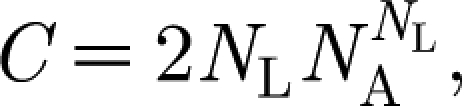
 is approximately twice that of the model with no transient immunity.

## 3. System dynamics

Simulations were undertaken for two scenarios of pathogen genome complexity, one assuming seven loci and three alleles allowing for 3^7^=2187 strains in total, and the other assuming six loci and three alleles allowing for 3^6^=729 strains.

We continue to assume that the cross-immunity function *γ*(*d*) has the form given in equation (2.3). We focus on examining dynamics for large *a* (*a*=0.2–0.5) corresponding to three or six allele substitutions at different loci being required to completely escape prior immunity. Cross-immunity generated by one strain against strains only one allele different from that strain is very high (*b*=0.95 or 0.9). These parameters are chosen to be ‘influenza-like’, in that they might be expected to maximize diversifying selection. The birth/death rate and mutation rate of the virus are the same as for the deterministic model (*μ*=0.014 per year and *m*=10^−4^ per year). For all simulation runs, we assume that initially there is only one strain and after a period of approximately five months introduce another strain, which contains only two novel alleles. We equilibrate the model for 100 years (chosen by observing the period required for model equilibration) before analysing 200 years of model output.

**Figure 1:**
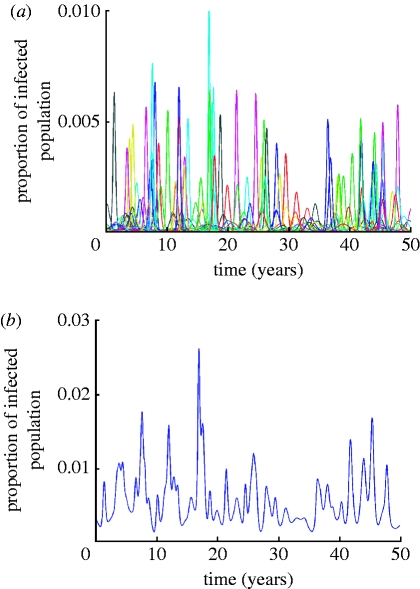
(*a*) Example time series for the proportion of infected in 1 billion population (virus genotype consists of six loci and three alleles). Curves of different colour show the dynamics of individual virus strains. (*b*) Temporal dynamics of the proportion of infected with all prevalent strains. The following parameter values were used: *a*=0.5, *b*=0.9, *R*_0_=2, *σ*=50.

The dynamics of the system is essentially different from the one of the deterministic model we studied before. In particular, regular periodic dynamics and equilibria are superseded by epidemic oscillations with widely varying amplitudes. An example time series for the proportion of infected population is portrayed in figure 1 *a*,*b*. The average prevalence of infection grows as cross-immunity becomes more specific (i.e. for increasing *a*).

**Figure 2:**
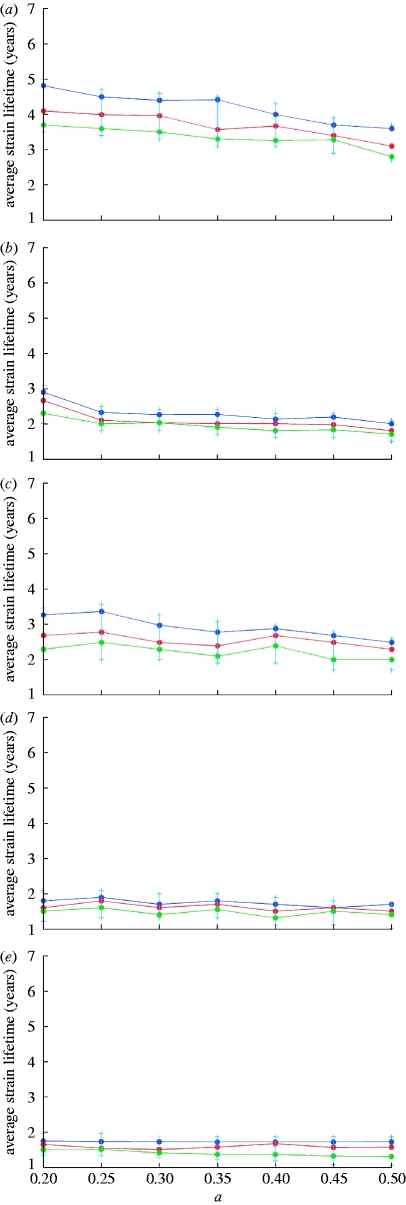
Average strain lifetime as a function of the cross-immunity parameter *a* for a genotype with seven loci and three alleles. *R*_0_ is taken to be (*a*,*b*) 2, (*c*,*d*) 4 and (*e*) 6. Maximum cross-immunity value, *b*, is 0.95 in all the cases. Results for a recovery rate of (*a*,*b*) *σ*=50 per year and (*c–e*) for *σ*=100 per year are shown (population size: blue dots, 1 000 000 000; red dots, 300 000 000; green dots, 90 000 000).

**Figure 3:**
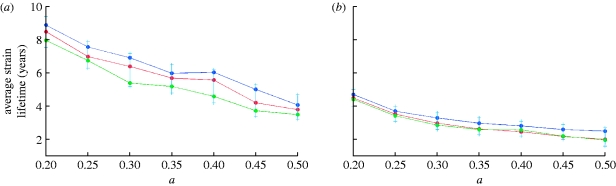
Average strain lifetime as a function of the cross-immunity parameter *a*, for *R*_0_=2, *b*=0.9 and a genotype with six loci and three alleles. Results for a recovery rate of (*a*) *σ*=50 per year and (*b*) *σ*=100 per year are shown (population size: blue dots, 1 000 000 000; red dots, 300 000 000; green dots, 90 000 000).

Mean strain lifetimes for the fully deterministic model are effectively infinite since only an intrinsic fitness deficit (lower *R*_0_) can cause extinction. Allowing for stochastic extinction and mutation processes drastically alters this picture, leading to mean strain lifetimes of a few years. Figure 2 *a–e* portrays dependences of mean strain lifetimes on the cross-immunity parameter *a* for the genotype consisting of seven loci and three alleles and two different values of generation time (specified by 1/*σ*) and three values of *R*_0_. The maximum value of cross-immune response, *b*, is fixed and equal to 0.95. Mean strain lifetimes for a genotype with six loci and three alleles and *b*=0.9 are plotted in figure 3 *a*,*b*. Colour dots in these graphs correspond to the results of single model runs and solid lines indicate general trends depending on the value of the cross-immunity parameter *a*. Owing to increasing prevalence and growing competition between strains, average strain lifetimes shorten as the cross-immune response becomes more specific (i.e. for increasing *a*). At large maximum values of cross-immune response (*b* close to 0.95), this dependence is fairly weak (figure 2 *a–e*) but becomes more distinct at *b*⪷0.9 (figure 3 *a*,*b*). Strain lifetimes also shorten (although only slowly) with decreasing population size (note that the extinction threshold for a strain is the prevalence of infection dropping below 1/*N*, where *N* is host population size). As expected, the lifetime of strains scales linearly with the generation time 1/*σ*, although this is only true where *σ*≫*μ*. Increasing *R*_0_ at fixed *σ* leads to a higher competition between strains thus also shortening their lifetimes. The dependence of strain lifetimes on *R*_0_ appears to be almost linear within the studied parameter range.

More insight can be obtained from examining the distribution of strain lifetimes. The cumulative distribution function *F*_s_(*t*) is defined as the percentage of strains having lifetimes less than or equal to *t* and density function *f*_s_(*t*) is the percentage of strains whose lifetimes fall within the interval (*t*,*t*+*δt*), where *δt* is a constant, which we assume to be equal to 1 year. For examples of *F*_s_(*t*) and *f*_s_(*t*) plotted for a population of 300 million, see figure 10 *a*,*b*. Maximum strain lifetimes can be rather long: approximately 100 years when *R*_0_=2 and *σ*=50 per year, halving when the generation time is halved (*σ*=100 per year). However, the proportion of strains surviving so long is very low. The vast majority of strains have lifetimes of a few years—when *σ*=50 per year, approximately 84 per cent of strains survive less than 5 years for a population of 1 billion, rising to 88 per cent for a population of 90 million. For *σ*=100 per year, the percentage going extinct in under 5 years rises to 95 per cent even in populations of 1 billion.

**Figure 4:**
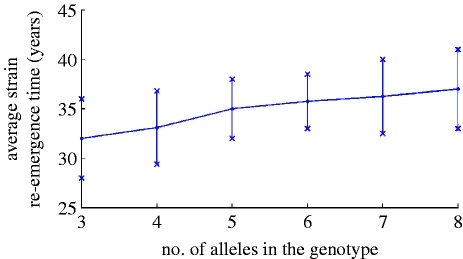
Average re-emergence time of virus strains for a genotype containing four loci plotted for 300 million population, *R*_0_=2, *a*=0.5, *b*=0.95 and *σ*=100 per year.

Since the genotype structure used in the model allows only for a limited number of strains, their stochastic re-emergence is inevitable. We examined how the average time of strain re-emergence depends on the number of alleles assumed per locus for a four loci antigenic ‘genome’. Figure 4 shows a weak linear trend when the number of alleles is varied between 3 and 8 (81–6561 different strains).

**Figure 5:**
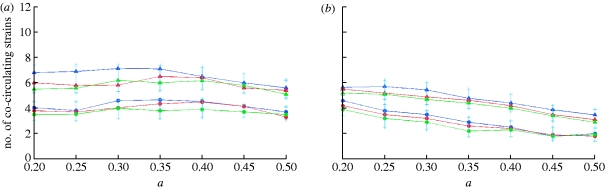
Prevalence-weighted numbers of strains circulating yearly as a function of the cross-immunity parameter *a*, for *R*_0_=2, (*a*) *b*=0.95 and (*b*) *b*=0.9. Colour triangles correspond to the results for a recovery rate of *σ*=50 per year and circles to the results for *σ*=100 per year. (*a*) Results for a seven-loci, three-allele genotype and (*b*) for a six-loci, three-allele genotype are shown (population size: blue, 1 000 000 000; red, 300 000 000; green, 90 000 000).

**Figure 6:**
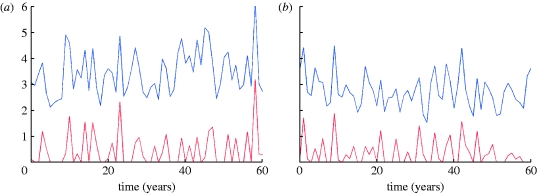
Time dependence of the prevalence-weighted number of co-circulating strains (blue curve) and strain extinction rate (red curve). Both graphs are for 300 million population, *R*_0_=2, *b*=0.9 and a genotype of six loci and three alleles. Recovery rates of (*a*) *σ*=50 per year and (*b*) *σ*=100 per year.

To characterize the overall diversity of the pathogen population through time, we use a frequency-weighted measure of diversity, 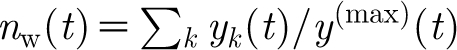 where *y*_*k*_(*t*) is the proportion of infected with strain *k* at time *t*, and *y* ^(max)^(*t*) is the maximum of the set {*y*_*k*_(*t*)}. If all strains have equal prevalence, this expression just reduces to the number of strains. Simulation shows that *n*_w_ depends weakly on population size and the cross-immunity parameter *a*, but is more strongly affected by the recovery rate *σ* and genome complexity (figure 5 *a*,*b*). Varying *R*_0_ with fixed *σ* does not have a significant effect on *n*_w_ in the range *R*_0_∈[2,6]. However, there is a strong negative correlation between diversity and generation time, as determined by *σ* (figure 6 *a*,*b*).

**Figure 7:**
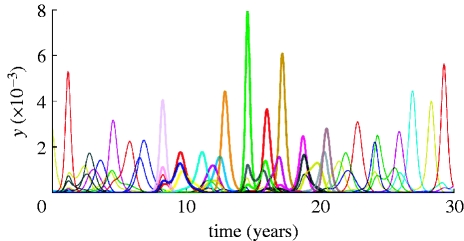
Sample time series for the proportion of infected population (300 million population model, *R*_0_=2, *σ*=50 per year; cross-immune response function, *γ*(*d*), is defined by the parameters *a*=0.45 and *b*=0.9). A few peaks corresponding to different strains are plotted in bold showing sequential strain replacement. The duration of strain-transcending immunity is 0.5 year.

We now consider the impact of transient strain-transcending immunity on these dynamics. Figure 7 shows temporal dynamics of strain-specific infection prevalence for a pathogen with six antigenic loci, each with three alleles (i.e. 729 strains in total). Results are shown for mean duration of strain-transcending immunity of 0.5 year. The series of peaks plotted in different colours in bold demonstrates sequential strain replacement occurring on a short time scale (1–3 years), a phenomenon typical for influenza virus. On a longer time scale (more than 50 years), however, re-emergence of strains is inevitable due to the limited total number of strains being modelled.

**Figure 8:**
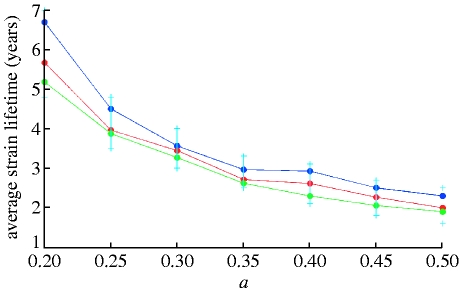
Dependence of average strain lifetimes on cross-immunity parameter *a*. The duration of strain-transcending immunity is 0.5 year, *R*_0_=2, *σ*=50 per year and *b*=0.9 (population size: blue dots, 1 000 000 000; red dots, 300 000 000; green dots, 90 000 000).

**Figure 9:**
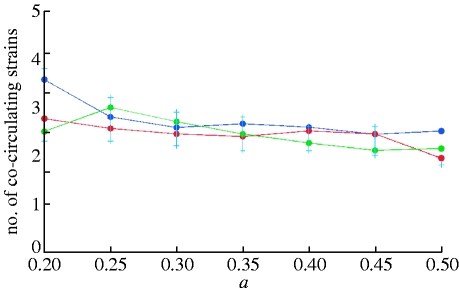
Dependence of average number of co-circulating strains on cross-immunity parameter *a*. The duration of strain-transcending immunity is 0.5 year. Model parameters are the same as in figure 8 (population size: blue dots, 1 000 000 000; red dots, 300 000 000; green dots, 90 000 000).

**Figure 10:**
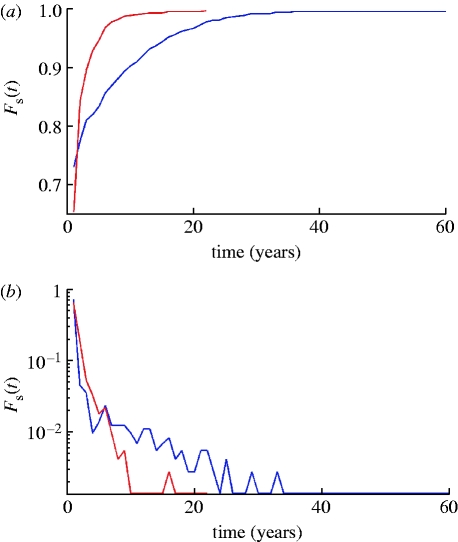
Comparison of strain lifetime distribution, *F*_s_(*t*) (*a*), and density, *f*_s_(*t*) (*b*), functions for models with (red curves) and without (blue curves) transient strain-transcending immunity. Duration of transient immunity is taken to be 0.5 year. Other model parameters are the same as in figure 9.

Transient non-specific immunity also leads to shortening average strain lifetimes (figure 8) and lower diversity (figure 9). For the model with six-month long transient immunity, the reduction in strain lifetimes and diversity is 1.5- to twofold, compared with the model with the same base parameters but without non-specific immune response. Examining the distribution of strain lifetimes, figure 10 *a*,*b* shows examples of *F*_s_(*t*) and *f*_s_(*t*) plotted for a population of 300 million for the models both with (red curve) and without (blue curve) transient immunity. Maximum strain lifetimes vary from 20 to 45 years depending on the duration of temporary immunity (the longer the duration the shorter is the maximum strain lifetime) and are generally 1.5- to 2.5-fold shorter than in the absence of transient immunity. The number of strains surviving so long is very few and all have very distinctive and similar dynamics: after an initial epidemic peak, a deep prevalence trough occurs that can last approximately one decade. During this trough, strains do not go completely extinct but, infecting a very small proportion of population (10^−6^–10^−5^), survive to give rise to a new epidemic peak or series of peaks once population immunity to strain wanes. It is arguable that improving model realism via the inclusion of spatial heterogeneity and seasonality of transmission into the model would eliminate such long-lived strains, but in order to maintain model simplicity we do not explore such modifications here.

**Figure 11:**
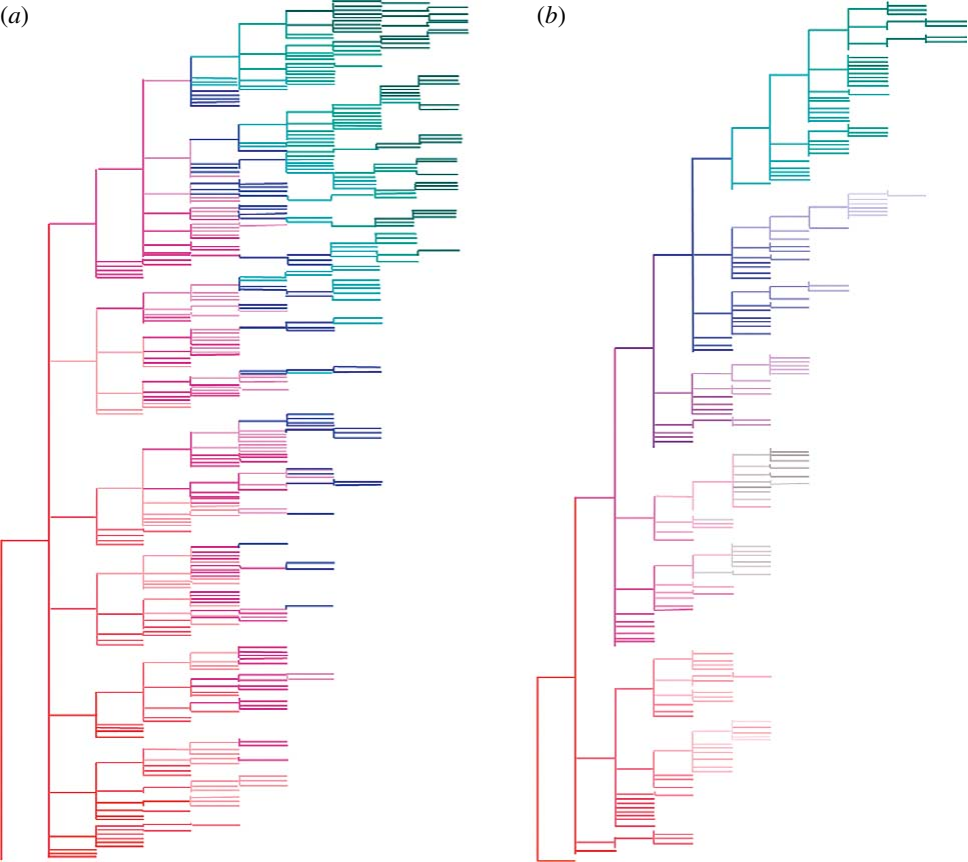
Comparison of example phylogenetic trees constructed from 50 years of output from the models (*a*) without transient non-specific immunity and (*b*) with transient immunity. Duration of transient immunity is 0.5 year and other model parameters are the same as in figure 9. Colour segments indicate times when strains are first found in circulation.

**Figure 12:**
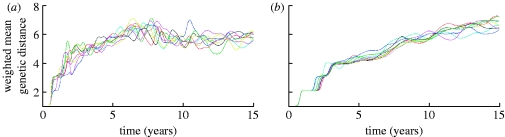
Graphs showing genetic drift for a model (*a*) without transient non-specific immunity and (*b*) with transient immunity plotted for a total of nine runs. For both models, 10-loci, two-allele genotype is considered and the other parameters are the same as in figure 9.

Phylogenetic trees generated from 50 years of model output also indicate that adding transient non-specific immunity gives more ‘flu-like’ evolutionary dynamics (figure 11 *a*,*b*), with a pattern of sequential replacement of fairly short-lived lineages seen (compared with much greater standing diversity for the model without transient immunity). These dynamics can also be seen by examining the degree of genetic drift over time. Figure 12 *a*,*b* shows the number of genetic changes accumulated by strains over time for a number of runs of the models without and with strain-transcending immunity. In the absence of non-specific immunity, strain diversification occurs quite rapidly and in just 3–4 years the genetic drift curve reaches a plateau (caused by the finite numbers of strains in the model), whereas, for the model with non-specific immunity, the drift curve is more linear over time, similar to the one observed for influenza A. Eventually, even in that case the curve saturates due to the limited number of strains possible in the model. This is a common drawback for all models with low genotype complexity (e.g. Girvan *et al*. (2002) where a 32 bit string model was studied).

In summary, including transient non-specific immunity results in a model that better matches the observed epidemiological and evolutionary dynamics of human influenza A viruses, with realistically low levels of strain diversity and reasonable strain lifetimes, together with sequential strain replacement. However, even the semi-stochastic model without transient immunity is a closer match to influenza dynamics than the purely deterministic formulation of the same model (Minayev & Ferguson 2008).

## 4. Conclusions

This paper has introduced a new class of multi-strain disease models that retain the relatively parsimonious compartmental structure of previous models (Andreasen *et al*. 1997; Gupta *et al*. 1998; Minayev & Ferguson 2008) but accounts for finite population sizes by explicitly modelling strain extinction, and captures the stochasticity of the mutation process. The dynamics of these ‘semi-stochastic’ models are strikingly different from those of the entirely deterministic model, with large amplitude limit cycles and chaotic behaviour typically being replaced by oscillatory dynamics with a limited number of dominant strains circulating at any one time. The maximum lifetime of a strain can be long—with the 99th percentile lifespan being approximately 40 years (figure 10 *a*,*b*, blue curves)—but the majority of strains go extinct within 5 years. Transient non-specific immunity shortens strain lifespan still further, reducing the upper 99th percentile lifetime to approximately 10 years (figure 10 *a*,*b*, red curves).

Overall, we assessed the influence of population size, generation time, genome complexity and cross-immunity on system dynamics. As expected, average strain lifetime and frequency-weighted diversity depend approximately logarithmically on host population size, with both variables being more strongly affected by pathogen generation time (diversity being inversely proportional to generation time) and the number of antigenic loci and alleles assumed.

Our analyses demonstrate that the processes underlying inter-strain competition and the complex epidemic dynamics resulting from such competition are strongly shaped by the processes that generate and remove strains—namely mutation and extinction. This is especially true for diseases with short generation times and high mutation rates, such as influenza. Indeed, our results cast doubt on the hypothesis proposed in Recker *et al*. (2007) to explain the antigenic evolution of influenza A. This is because the diversity of antigenic types circulating at any time in the model proposed in that paper was restricted assuming a very small number of genetic loci determined influenza antigenicity and that no mutation occurred. Increasing the number of antigenic loci and alleles to more reasonable levels and incorporating strain extinction and stochastic mutation give very different dynamics, as illustrated in this paper. Strain recycling then only occurs by strains being regenerated via mutation (due to the finite size of the modelled antigenic genome)—a process that has never been observed in reality. Indeed, recent data from haemagglutinin inhibition tests comparing strains spanning the entire evolutionary history of H3N2 find show no evidence of antigen recycling (Smith *et al*. 2004).

Our results demonstrate that incorporation of transient strain-transcending immunity reduces diversity and strain lifetime giving dynamics that more closely resembles those of human influenza A (e.g. sequential replacement of dominant strains) than models that do not include such immunity—in agreement with prior simulation studies (Ferguson *et al*. 2003). However, the models explored in this paper do not incorporate spatial heterogeneity or seasonality in transmission, both factors that have a substantial effect on strain establishment, competition and extinction (Rambaut *et al*. 2008; Russell *et al*. 2008). This limits the extent to which results can be quantitatively compared with epidemiological or evolutionary data for human influenza.

The semi-stochastic model we have developed here has the advantage of having relatively low computational complexity compared with full individually based simulations (Ferguson *et al*. 2003; Koelle *et al*. 2006), which makes rigorous exploration of parameter space more feasible. The key weakness of the model structure is that it allows only rather limited antigenic diversity to be considered as model dimensionality grows exponentially with the number of antigenic loci and as a power function of the number of alleles assumed. For that reason, extinct strains can randomly re-emerge in short time (25–50 years for the genotype sizes explored here), which somewhat limits the realism with which this type of model (whether stochastic or deterministic) can describe influenza evolution. The same shortcoming also applies to other compartmental frameworks, such as status-based models (Gog & Grenfell 2002). However, despite this limitation, the models developed here are potentially useful as descriptions of the diversity and evolution of other pathogens, most notably bacteria such as *Neisseria meningitidis* (Gupta *et al*. 1996) and viruses such as dengue (Ferguson *et al*. 1999). Extending the current framework to include recombination might also allow relatively parsimonious description of the population-level evolution of macroparasite infections, such as malaria.
